# Ozone-Based Eye Drops Activity on Ocular Epithelial Cells and Potential Pathogens Infecting the Front of the Eye

**DOI:** 10.3390/antiox10060968

**Published:** 2021-06-16

**Authors:** Roman Paduch, Teresa Urbanik-Sypniewska, Jolanta Kutkowska, Tomasz Chorągiewicz, Anna Matysik-Woźniak, Sandrine Zweifel, Aleksandra Czarnek-Chudzik, Wojciech Załuska, Robert Rejdak, Mario Damiano Toro

**Affiliations:** 1 Department of Virology and Immunology, Faculty of Biology and Biotechnology, Institute of Biological Sciences, Maria Curie-Skłodowska University, Akademicka 19, 20-033 Lublin, Poland; 2 Department of General and Pediatric Ophthalmology, Medical University of Lublin, Chmielna 1, 20-079 Lublin, Poland; tomekchor@wp.pl (T.C.); annawozniak@umlub.pl (A.M.-W.); robertrejdak@yahoo.com (R.R.); mario.toro@usz.ch (M.D.T.); 3 Department of Genetics and Microbiology, Maria Curie-Skłodowska University, Akademicka 19, 20-033 Lublin, Poland; teresa.urbanik-sypniewska@poczta.umcs.lublin.pl (T.U.-S.); jolanta.kutkowska@poczta.umcs.lublin.pl (J.K.); 4 Department of Ophthalmology, University of Zurich, 8091 Zurich, Switzerland; sandrine.zweifel@usz.ch; 5 Department of Diagnostic and Microsurgery of Glaucoma, Medical University of Lublin, Chmielna 1, 20-079 Lublin, Poland; olaczarnek@poczta.fm; 6 Department of Nephrology, Medical University of Lublin, Jaczewskiego 8, 20-090 Lublin, Poland; wojciech.zaluska@umlub.pl

**Keywords:** ozone-based drops, toxicity, bacteria, conjunctival epithelium, corneal epithelium, cell culture in vitro

## Abstract

Confirmation of the biological effectiveness of new ophthalmic preparations introduced in the market is an important element in maintaining the safety of using this type of medications. This study aimed to investigate the activity of Ozodrop^®^ on human corneal and conjunctival epithelial cells, as well as its antibacterial and antifungal activity. Cytotoxicity analyses of ocular surface epithelial cells were performed in vitro by MTT (3-(4,5-Dimethylthiazol-2-yl)-2,5-Diphenyltetrazolium Bromide) and Neutral Red uptake assays. The level of nitric oxide released by the cells was assessed by the Griess method. The reduction of the DPPH (2,2-diphenyl-1-picrylhydrazyl) free radical by the tested formulation was analyzed. Microbiological tests were also performed. It was found that the Ozodrop^®^ preparation exhibited biological activity, but was less active than the reference antibiotics and the anti-yeast agent. The cytotoxic activity of the Ozodrop^®^ formulation was dependent on the time of cell exposure to it. No toxic effect was observed in the short-term, for up to 3 h. It appeared after 24 h of exposure of the cells to the preparation. The drops showed antioxidant activity in the specified concentration range. They also stimulated the release of nitric oxide, mainly by corneal epithelial cells. The Ozodrop^®^ formulation exhibits biological activity that can be considered useful in the treatment of infections in the front part of the eye.

## 1. Introduction

Ozone (O_3_) is a triatomic allotrope form of oxygen with a dynamic unstable structure [1,2]. This molecule is more and more widely described as an agent expressing antiviral, antibacterial, antifungal, and anti-inflammatory activities, an immunomodulating factor, as well as a redox state modulator and an agent with cytoprotective properties [1,3]. It owes this type of activity to a strong oxidative power that does not induce resistance. However, ozone also has adverse effects. According to a WHO (World Health Organization) report, prolonged O_3_ exposure may lead to chronic damage to the human lung, inflammation, incidence of asthma, problems with lung function and growth, or even lung cancer [4] Hence, there is ongoing search for the best form of application of this molecule to eliminate the side effects and maintain the healing effect. In the field of ophthalmic research and applications against infectious conditions of the eye, ozonide eye drops have been introduced for ocular surface disinfection [5]. In this type of preparation, ozone is stabilized by forming ozonized oil as a product of the reaction of gaseous ozone with unsaturated fatty acids. One approved formulation for ophthalmic use containing ozonized oil in sunflower liposomes is Ozodrop^®^ (FB Vision, San Benedetto del Tronto, Italy) [2,5]. This preparation has been designed to facilitate the healing of damage to the front of the eye and to protect the eye against potential infections of various etiology. In addition, the emergence of resistance to commonly used antibiotics prompts researchers to look for new solutions related to the factors protecting the eye against infection and its consequences, including blindness. It has been pointed out that ozonated oils should not adversely affect biological structures, either at the cellular or the whole tissue level. The ozone contained in Ozodrop^®^ has a direct cytotoxic effect on pathogens such as bacteria, through oxidation, leading to the lysis and decay of pathogenic organisms. Moreover, it has been suggested that the use of these drops may adapt the eye tissue to them and thus make the eye surface resistant to extrinsic free radicals [5,6].

Eye infections can be caused by bacteria, fungi, or viruses. Bacterial ocular infections include conjunctivitis, keratitis, endophthalmitis, blepharitis, and orbital cellulitis. *Staphylococcus aureus*, *Pseudomonas aeruginosa*, and *Escherichia coli* are associated with many types of eye infections, but primarily with blepharitis, conjunctivitis, dacryocystitis, and keratitis [7]. *S*. *aureus* and *P*. *aeruginosa* often cause contact-lens-associated infectious keratitis. The opportunistic pathogen *Moraxella catarrhalis*, which commonly affects immunocompromised individuals, can also be an etiological factor of keratitis [8]. Fungal ocular infections can be caused by filamentous fungi such as *Fusarium* spp. and *Aspergillus* spp. and the yeast-like fungi *Candida* spp., including *C*. *albicans* [5]. Increased bacterial antibiotic resistance contributes to the use of medicines based on natural products, such as ozonized oil, which have a wide spectrum of antimicrobial effects for therapeutic applications.

The aim of the present study was to verify the biological activity of Ozodrop^®^ on human cells of the conjunctival and corneal epithelia, as well as to assess the potential antibacterial effectiveness of the preparation against the most common pathogens that infect or may pose a threat to the front of the eye.

## 2. Materials and Methods

### 2.1. Cell Lines

A normal human corneal epithelial cell line, 10.014 pRSV-T (ATCC No. CRL-11515), and a human conjunctival epithelial cell line, HC0597 (LGC Standards, Teddington Middlesex, UK), were used. The cells were cultured as monolayers in 25 cm^2^ culture flasks (NuncTM, Roskilde, Denmark) coated with PureColTM ultrapure collagen type I (INAMED Biomaterials, Fremont, CA, USA), at 3.1 mg/mL concentration (about 12 μg/cm^2^). The cell lines were maintained in a defined keratinocyte serum-free medium (K-SFM) (GibcoTM, Paisley, UK), supplemented with 75 μg/mL endothelial cell growth factor (ECGF) (Sigma, St. Louis, MO, USA), 0.05 mg/mL bovine pituitary extract (BPE) (Gibco), 500 ng/mL hydrocortisone (Sigma, St. Louis, MO, USA), 0.0005 mg/mL bovine insulin (Gibco), and antibiotics (100 U/mL penicillin, 100 μg/mL streptomycin) (Sigma, St Louis, MO, USA), in a humidified atmosphere with 5% CO_2_, at 37 °C.

### 2.2. Microbial Strains

The activity of Ozodrop was assessed against the Gram-positive bacteria *Staphylococcus aureus* ATCC No. 29213, *S*. *aureus* ATCC No. 43300 (a methicillin resistant strain, MRSA), *Staphylococcus epidermidis* ATCC No. 14990 (a coagulase negative strain, CNS), the Gram-negative bacteria *Moraxella catarrhalis*, *Pseudomonas aeruginosa* ATCC No. 27683, *Escherichia coli* ATCC No. 25922, and the yeast strain *Candida albicans* (a clinical isolate).

### 2.3. Experimental Design

The cells were cultured in 24-well (nitric oxide level and staining) or 96-well (viability) plates (Nunc^TM^). The cells were seeded on the well bottom at a density of 1 × 10^5^ cells/mL. After 24 h of inoculation, the medium was changed to one containing the following dilutions of Ozodrop^®^ (FB Vision, San Benedetto del Tronto, Italy):

1:200 (0.5% of the original starting concentration of Ozodrop^®^)

1:100 (1% of the original starting concentration of Ozodrop^®^)

1:40 (2.5% of the original starting concentration of Ozodrop^®^)

1:20 (5% of the original starting concentration of Ozodrop^®^)

1:13.3 (7.5% of the original starting concentration of Ozodrop^®^)

1:10 (10% of the original starting concentration of Ozodrop^®^)

1:8 (12.5% of the original starting concentration of Ozodrop^®^)

1:6.7 (15% of the original starting concentration of Ozodrop^®^)

Dilutions were made in the culture medium.

After cell induction, the culture was conducted for a further 1, 3, and 24 h. Next, cytotoxicity (NR) and metabolic activity (MTT) were analyzed. Moreover, culture supernatants were collected and analyzed for the levels of nitric oxide (NO). The individual dilutions of Ozodrop^®^ were analyzed in terms of their ability to reduce free oxygen radicals (DPPH).

### 2.4. Neutral Red (NR) Uptake Assay

The cells (1 × 10^5^ cells/mL) were grown in 96-well multiplates, for 24 h, in 100 μL of CS-C culture medium, with the supplements and appropriate Ozodrop^®^ dilutions. After 1, 3, and 24 h of incubation, the medium was discarded and 0.4% NR (Sigma, St. Louis, MO, USA) solution medium was added to each well. The plate was incubated for 3 h at 37 °C in a humidified 5% CO_2_/95% air incubator. After incubation, the dye-containing medium was removed, the cells were fixed with 1% CaCl_2_ in 4% paraformaldehyde, and the incorporated dye was solubilized using 1% acetic acetate in a 50% ethanol solution (100 μL). The plates were gently shaken for 20 min at room temperature, and the extracted dye absorbance was measured spectrophotometrically at 540 nm, using an E-max Microplate Reader (Molecular Devices Corporation, Menlo Park, CA, USA).

### 2.5. MTT Assay

Cell viability after incubation with appropriate Ozodrop^®^ dilutions was determined in a standard spectrophotometric 3-(4,5-dimethylthiazole-2-yl)-2,5-diphenyltetrazolium bromide (MTT) assay. The cells (1 × 10^5^ cells/mL) grown in the 96-well multiplates in 100 μL of the culture medium were incubated for 1, 3, and 24 h, with the ophthalmic drops. Next, an MTT solution (5 mg/mL, 25 μL/well) (Sigma, St. Louis, MO, USA) was added and further incubation was conducted for the next 3 h. The yellow tetrazolium salt was metabolized by viable cells to purple formazan crystals. The reaction was catalyzed by mitochondrial succinyl dehydrogenase. The crystals were solubilized overnight in a 10% sodium dodecyl sulfate (SDS) in a 0.01 M HCl mixture. The product was quantified spectrophotometrically through absorbance measurement at 570 nm wavelength, using an E-max Microplate Reader (Molecular Devices Corporation, Menlo Park, CA, USA).

### 2.6. Nitric Oxide (NO) Measurement

Nitrate, i.e., a stable end product of NO, was determined in the culture supernatants with a spectrophotometric method, based on the Griess reaction. In brief, the cells were induced and incubated for 24 h, with appropriate dilutions of the Ozodrop^®^. Thereafter, 100 μL of the supernatant was plated in 96-well flat-bottomed plates, in triplicates, and incubated with 100 μL of Griess reagent (1% sulphanilamide/0.1% N-(1-naphthyl) ethylenediamine dihydrochloride) (Sigma, St. Louis, MO, USA) in 3% H_3_PO_4_ (POCH Gliwice, Poland), for 10 min at room temperature. The optical density was measured at 550 nm using a microplate reader (Molecular Devices Corp., Emax, Menlo Park, CA, USA). A standard curve was achieved using 0.5–25 μM sodium nitrite (NaNO_2_) for calibration.

### 2.7. DPPH Free Radical Scavenging Test

Free radical scavenging activity was measured by the 1,1-diphenyl-2-picrylhydrazyl (DPPH) assay. This method is based on the ability of antioxidants to reduce the stable dark violet radical DPPH (Sigma-Aldrich Co., St. Louis, MO, USA) to the yellow-colored diphenyl-picrylhydrazine. In brief, 100 μL of DPPH solution (0.2 mg/mL in ethanol) was added to 100 μL of the appropriate dilutions of the Ozodrop^®^ and standards. Trolox (Sigma-Aldrich Co., St. Louis, MO, USA) at increasing concentrations (1–50 μg/mL) was used as a standard for free radical scavenging activity. After 20 min incubation at room temperature, the absorbance of the solution was measured at 515 nm. The lower the absorbance, the higher the free radical scavenging activity of the Ozodrop^®^ dilution. The activity of each dilution was determined by comparing its absorbance with that of a control and standards.

The capability to scavenge DPPH radical was calculated by the following formula:

DPPH scavenging effect (%) = [(Xcontrol − XOzodrop^®^ dilution /Xcontrol) × 100] (1)

where Xcontrol is the absorbance of the control and XOzodrop^®^ dilution is the absorbance in the presence of appropriate Ozodrop^®^ dilution.

### 2.8. May–Grünwald–Giemsa (MGG) Staining

The MGG staining enable us to visualize the morphological changes in cells induced by Ozodrop^®^.

The cells at a density of 1 × 10^5^ cells/mL were cultured in Petri dishes (35 mm). After 24 h od inoculation, the medium was changed, and the cells were incubated with Ozodrop^®^ for 24 h. After incubation, the cells were fixed with methanol for 5 min and stained with the May-Grünwald dye diluted in an equal volume of water for 2 min. Thereafter, the dye was removed and the previously diluted Giemsa stain (1 vol. Giemsa: 19 vol. water) was added for 20 min. The dishes were rinsed three times with distilled water and dried. The observation was performed under a light microscope (Olympus BX51, Tokyo, Japan).

### 2.9. Determination of Minimum Inhibitory Concentration (MIC)

The antimicrobial activity of Ozodrop^®^ was determined by broth microdilution in 96-well microplates, according to the standard method, as detailed in [9]. The concentration of ozonized sunflower oil was 0.5% in the finished formulation, corresponding to 5 mg/mL. Ozodrop^®^ was serially diluted in Mueller Hinton Broth (MHB), from a starting concentration of 250 to 0.25 (μg/mL) of ozonized sunflower oil.

Gentamicin (Polfa Warszawa), Tobramycin (Polfa Warszawa), and Levofoxacin (Adamed) were used as antibiotic reference for the bacteria, and itraconazole (Janssen-Cilag International) was used for the yeast. Stock solutions of the reference drugs were prepared at 10-fold the final concentration. Two-fold dilutions ranging from 256 to 0.015 μg/mL were prepared in MHB.

The microtubes were inoculated with a standardized bacterial or fungal suspension of 1 × 10^5^ CFU/mL. Microorganism growth was detected spectrophotometrically with the microplate reader ASYS UVM 340 (Biogenet), after 18-h incubation of bacteria or 24-h incubation of yeast at 37 °C. The minimum inhibitory concentration (MIC) was defined as the lowest concentration of a drug inhibiting the growth of the tested microorganism, in comparison to a drug-free growth control.

After MIC determination, aliquots of 50 μL from all wells that showed no bacterial growth were spread on Mueller Hinton agar plates without drugs, and were incubated for 24 h at 37 °C. Then, the number of colonies was counted. The concentration of a drug in which no colonies had formed (<10 colonies) was considered as the minimum microbicidal concentration (MMC). Each experiment was repeated three times.

### 2.10. Statistical Analysis

The results are presented as means ± SD of three independent experiments (*n* = 3). The data were analyzed using one-way analysis of variance ANOVA, followed by Dunnett’s multiple comparison post-hoc test. Differences were considered significant at *p* ≤ 0.05.

## 3. Results

### 3.1. General Data

In the present study, the potential cytotoxic activity (NR uptake assay), metabolic activity (MTT assay), and the nitric oxide (NO) level induced in human corneal and conjunctival epithelial cells by the Ozodrop^®^ ophthalmic solution were tested. Moreover, the capacity of these drops to scavenge free radicals was assessed by the DPPH reduction assay.

### 3.2. Cytotoxicity Assessment by Neutral Red (NR) Uptake Assay

**Figure 1:**
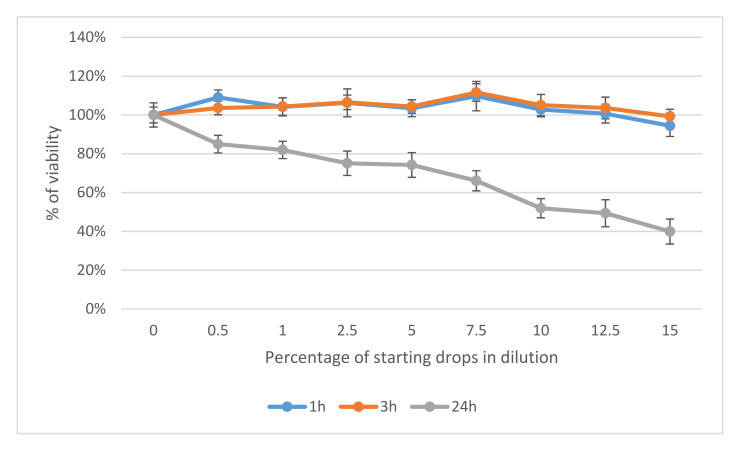
Effect of Ozodrop^®^ preparation on the viability of human corneal epithelium (pRSV-T) in vitro, after 1, 3, and 24 h of incubation. Cell viability was determined by means of neutral red cytotoxicity (NR) assay.

**Figure 2:**
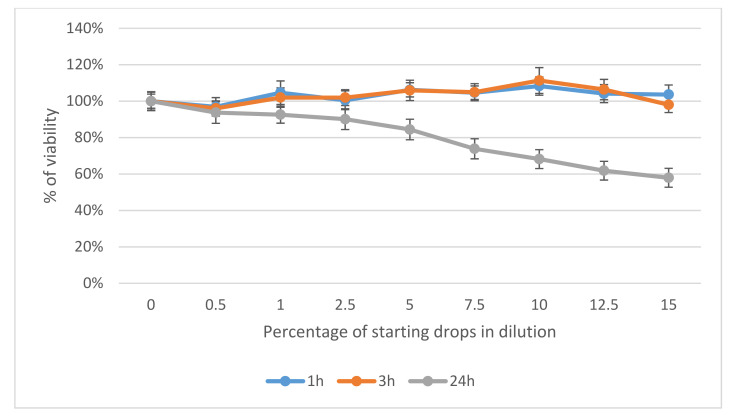
Effect of Ozodrop^®^ preparation on the viability of human conjunctival epithelium (HC0597) in vitro after 1, 3, and 24 h of incubation. Cell viability was determined by means of neutral red cytotoxicity (NR) assay.

Our results revealed that Ozodrop^®^ was safe after 1 h and 3 h of contact with human corneal and conjunctival epithelial cells. No toxic effects were observed. However, continuous contact of the drops with these cells for 24 h had a concentration-dependent toxic effect. Corneal epithelial cells showed a higher sensitivity than conjunctival cells. At a concentration of 15% of the original drops, a decrease in the viability of the cells was about 60%, as compared to the untreated control. In conjunctival cells, the same concentration reduced viability by 42% (Figure 1 and Figure 2).

### 3.3. Metabolic Activity Assessment by MTT Assay

**Figure 3:**
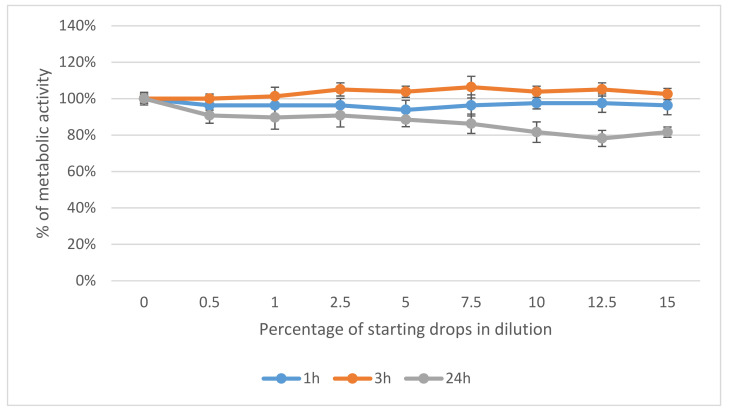
Effect of Ozodrop^®^ preparation on metabolic activity of human corneal epithelium (pRSV-T) in vitro, after 1, 3, and 24 h of incubation. Cell metabolic activity was determined by means of an MTT assay.

**Figure 4:**
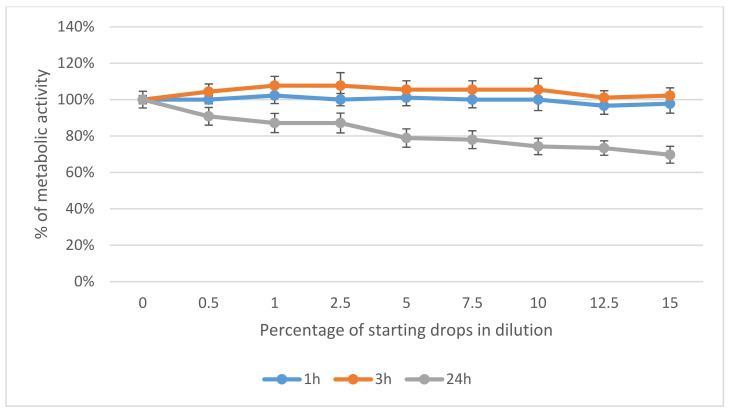
Effect of Ozodrop^®^ preparation on the metabolic activity of human conjunctival epithelium (HC0597) in vitro, after 1, 3, and 24 h of incubation. Cell metabolic activity was determined by means of an MTT assay.

After 1 h and 3 h of cell incubation with Ozodrop^®^, no metabolic disturbances were found. When cells were incubated for 24 h, Ozodrop^®^-concentration-dependent changes were observed. In this case, the human conjunctival epithelial cells revealed higher sensitivity than corneal cells. At a concentration of 15% of the original drops, the metabolic activity of conjunctival epithelial cells dropped by about 30%, as compared to the untreated control. In corneal cells, the same concentration reduced cellular metabolism by about 19.5% (Figure 3 and Figure 4).

### 3.4. Nitric Oxide (NO) Level

**Figure 5:**
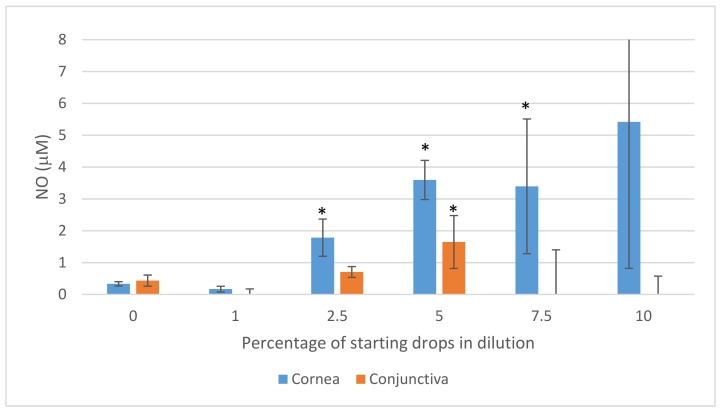
Nitric oxide (NO) secretion in a culture of human corneal and conjunctival epithelium, during 24 h of incubation with Ozodrop^®^ preparation. The concentration range of starting drops dilution was 0–10%. Analysis was performed using the Griess method. Columns and bars show the mean ± standard deviation (*n* = 3). * *p* ≤ 0.05—a culture of corneal or conjunctival epithelial cells treated with Ozodrop^®^ preparation, as compared to a non-treated culture control.

The application of Ozodrop^®^ differentially changed nitric oxide (NO) production by conjunctival and corneal epithelial cells, depending on the concentration used. The increasing concentration of the drops enhanced the release of NO by corneal epithelial cells. In conjunctival epithelial cells, the release of NO was increased by drops containing only up to 5% of the initial concentration of the active substance. Higher concentrations inhibited NO release in both cell types. Moreover, the concentrations of NO released after induction with the drops were higher in the corneal epithelial cells than in the conjunctival ones. Under control conditions, the two types of cells released similar amounts of NO (an average of 0.4 μM) (Figure 5).

### 3.5. Free Radical Scavenging Capacity Assessment by DPPH Reduction

The preparation we tested also showed the ability to reduce free oxygen radicals. However, this property was revealed only in the case of at least 10-fold dilutions of the starting preparation. Higher concentrations did not show this property. Starting from a 200-fold dilution of the original formulation (0.5% of the original droplet concentration), a gradual increase in the ability to reduce DPPH was observed. It reached a maximum with samples containing 2.5% to 7.5% of the original active substance. This value corresponded to the reducing activity of Trolox used at a concentration of about 7 μg/mL (Table 1).

### 3.6. May–Grünwald–Giemsa (MGG) Staining

**Figure 6:**
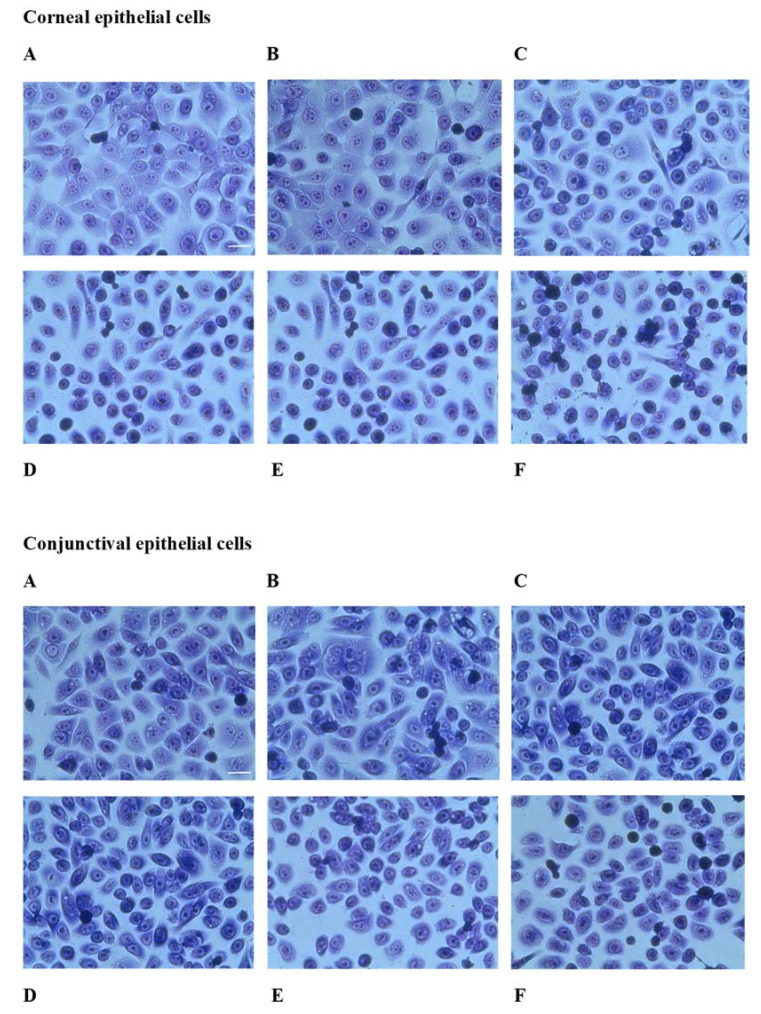
May–Grünwald–Giemsa (MGG) staining of the human corneal epithelial and conjunctival epithelial cells incubated with Ozodrop^®^ formulation for 24 h. (**A**) Control; (**B**) Ozodrop^®^ in a 1% concentration; (**C**) Ozodrop^®^ in a 2.5% concentration; (**D**) Ozodrop^®^ in a 5% concentration; (**E**) Ozodrop^®^ in a 7.5% concentration; and (**F**) Ozodrop^®^ in a 10% concentration. Bar = 20 μm. Magnification 200×.

After 1 h and 3 h of incubation of corneal and conjunctival epithelial cells with the preparation tested in dilutions of up to 10% of the stock solution, no morphological changes of the cells were found. After 24 h of incubation under control conditions, corneal epithelial cells had a cobblestone-type morphology. The cells were flattened on the culture surface and were in contact with the cell membranes. The cell nucleus and nucleoli were clearly marked. Stained cytoplasm was also clearly visible. Cells incubated with increasing concentrations of the original Ozodrop^®^ drops showed changes in morphology, starting from 2.5% of the stock solution of the preparation. Contraction and separation of the cells, limiting the surface of the visible cytoplasm and thus limiting the contact of the cells with the culture surface, was observed. A further increase in the concentration of the preparation led to a full contraction of the cytoplasm and merging of the nucleoli. At a concentration of 10% of the original drops, the cells became round and started to detach from the culture surface. Similar effects were observed when the conjunctival epithelial cells were analyzed (Figure 6).

### 3.7. In Vitro Antimicrobial Effects

The activity of the Ozodrop^®^ ozonized sunflower oil eye drops in liposome, against the microorganisms tested and the antibiotic susceptibility pattern, are presented in Table 2.

The strains most susceptible to Ozodrop^®^ were *S. aureus* and *C. albicans*, with MIC values of 31.25 μg/mL. The concentration inhibiting the growth of *S. epidermidis* and Gram-negative bacteria was two times higher at 62.5 μg/mL.

The value of MMC ranged from 62.5 μg/mL for *S. aureus* ATCC 29213 and *C. albicans*, to 125 μg/mL for the other strains.

The aminoglycoside antibiotics—gentamycin and tobramycin, fluoroquinolone levofloxacin (which is one of the most frequently used remedies for bacterial eye infections), and the azole antifungal agent—itraconazole were used as reference drugs. The bacterial strains tested were susceptible to all antibiotics used, while *C. albicans* showed resistance to itraconazole.

## 4. Discussion

In the present study, we aimed to evaluate the Ozodrop^®^ preparation, in the context of its toxicity towards the human cells of the anterior part of the eye (corneal and conjunctival epithelia) and to answer the question about the activity and effectiveness of this preparation against bacteria that poses a potential threat to this part of the eye. The experiments with corneal and conjunctival epithelial cells were carried out using the preparation, which was diluted in order to adjust its concentrations to in vitro conditions. The study shows that the contact time of the drops with the cells is important. The longer the cells were exposed to the preparation (24 h or longer), the more efficient the cytotoxic activity became, which could pose an in vivo hazard to eye tissues. However, a short time (up to 3 h) of contact of these drops with the epithelial surface did not have any adverse effect in the form of changes in cell morphology or a decrease in their viability. The purpose of introducing a short incubation time was to bring the analyses closer to the reality of the preparation being removed from the eye surface by tears or blinking. The lack of a toxic effect of the drops during this time indicates that the front part of the eye should not be damaged while using these drops. However, these are only in vitro tests, and so the results must be confirmed by further analyses in clinical conditions.

Research conducted with the participation of ozone showed that this molecule stimulates the production of nitric oxide (NO) and reactive forms of nitrogen by cells [10]. In our study, we observed an increase in the level of NO after introducing the ozone-based drops into the microenvironment of corneal and conjunctival epithelial cells. The increase was found mainly in the culture of corneal epithelial cells and depended on the concentration of the ozone-based drops. It can be suggested that, an elevated level of NO or oxidation products could be the cause of a significant decrease in the viability of the epithelial cells of the eye surface, after 24 h of incubation. Moreover, ozone exposure significantly affects the stability of cell membranes. This applies to both eukaryotic cells as well as bacterial and fungal ones. Both phospholipid and lipoprotein membrane components undergo changes in these conditions. The MTT assay showed a disturbance of cellular metabolism after 24-h exposure of cells to the Ozodrop^®^ preparation. This result may have been due to the fact that, after prolonged exposure, the drops exhibited cytotoxic activity not only against the cytoplasmic membrane, but also against the mitochondrial membranes, leading to the disruption of the latter’s fluidity, for example, as a result of lipid peroxidation. As a consequence, a disturbance in the functioning of the respiratory chain and cell death were observed. However, due to the secretion of tears and blinking, the prescribed preparation should not remain on the surface of the eye for a long time. The performed tests indicated a low toxicity of the drops for at least one hour of exposure to the corneal or conjunctival epithelium. Therefore, with additional dilution with tears and a shorter duration of residence on the eye surface, the preparation seems to be even safer to use. The study time we used was to some extent a limitation of the study. Therefore, further research in this direction should be carried out using a short exposure time in order to unequivocally confirm these assumptions. Recently, Celenza et al. 2020 also demonstrated the antifungal activity of Ozodrop^®^ against four species of *Candida* (*C*. *albicans*, *C*. *glabrata*, *C*. *krusei*, and *C*. *orthopsilosis*). The proposed mechanism of toxicity of the preparation was the generation of reactive oxygen species (ROS) and induction of alterations in mitochondrial membrane fluidity. This, in turn, may be associated with a high outflow of free radicals from damaged organelles and the associated disturbances in the microenvironmental level of nitric oxide [5]. Additionally, by stimulating the production of nitric oxide through epithelial cells, ozone-based drops also contribute to the development of local inflammation [11]. It has been pointed out that exposure of epithelial cells to ozone stimulates an increase in the expression of inflammatory markers related, inter alia, to the arachidonic acid pathway. Ozone stimulation of epithelial cells has been found to induce the production of inflammation-related proteins, including cytosolic phospholipase A2 (cPLA2) and cyclooxygenase-2 (COX-2) [12,13,14,15]. This stimulation can also lead to cell damage after long exposure to ozone-based drops. Indeed, changes in the metabolism and viability of cells after long-term exposure to ozone may be associated with the formation of toxic oxygen free radicals that can damage the structure of the epithelium [3]. It has also been shown that constituents of the particulate matter (PM) can generate reactive oxygen species, and consequently, induce oxidative stress. This has a significant effect on tear production, leading to dry eye symptoms. In this context, it has been observed that an increased concentration of ozone can reduce—while low molecular weight pollutants can increase—the production of tears [16]. However, there is also a positive side to the activity of ozone molecules. Free radicals appearing in the microenvironment after ozone exposure may exert a cytoprotective effect, mainly as modulators of the NF-κ/Nrf2 pathways, thus, maintaining the balance of pro-inflammatory factors [1]. Therefore, the activity of ozone should be considered in relation to the current condition of specific tissues and organs, their exposure to various concentrations of this molecule and the potential sensitivity and reactivity of the cells exposed to this factor. Hence, ozone can be viewed, depending on the specific conditions, to be either as an oxidative factor, or, when appropriately deployed, as a medicinal drug [17].

In our research, we have shown that in a certain concentration range, ozone-based drops have a strong antioxidant effect that can positively affect the eye cells. Therefore, time-limited exposure of the corneal epithelium and conjunctiva to ozone-based drops may have a beneficial impact on these cells, while limiting the development of pathogenic microflora, leading to infections of the anterior part of the eye. Our results show the antimicrobial efficacy of the Ozodrop^®^ ozonized sunflower oil eye drops in liposome against bacterial strains, including antibiotic-resistant *S*. *aureus* and *P*. *aeruginosa*, as well as the yeast *C*. *albicans*. Antimicrobial activity of a liposome-vehiculated ozonated oil against *S*. *aureus* and *P*. *aeruginosa* was observed by Cutarelli et al. [18]. Serio et al. [19] confirmed the antibacterial activity of ozonated sunflower seed oil (Oz.Or.Oil 30) against both Gram-negative (*E*. *coli* and *P*. *aeruginosa*) and Gram-positive (*S*. *aureus* and *Micrococcus luteus*) bacterial strains.

## 5. Conclusions

Summing up, the Ozodrop^®^ eye preparation exerts a beneficial effect, after a short time of exposure of human corneal and conjunctival epithelial cells to the preparation. Longer exposure times may have a toxic effect. The Ozodrop^®^ formulation can, therefore, be an interesting supplement to the compounds used so far, in order to establish an antioxidant/inflammatory balance in the front part of the eye. It has been shown that the preparation in the right concentration shows the ability to scavenge free oxygen radicals that can damage the cells of the eye surface. Moreover, the preparation tested in this study exhibits antibacterial and antifungal activities against potential pathogens that infect the surface of the eye. Although, Ozodrop^®^ was less active than the reference antibiotics and anti-yeast agent, it represents a potential alternative in the therapy or prevention of ocular infections. It seems that the drops can be safely administered as a supplement to the standard preparations used to protect the surface of the eye.

## Figures and Tables

**Table 1:** DPPH method—free radicals scavenging activity performed by different dilutions of the tested drops.

Drops Dilution	The Reduction Value that Corresponds to the Reduction by the Following Trolox Concentrations (μg/mL)
200× (0.5% from the stock solution)	4.576 ± 0.978
100× (1% from the stock solution)	5.905 ± 2.990
40× (2.5% from the stock solution)	7.685 ± 0.843
20× (5% from the stock solution)	7.871 ± 3.759
13.3× (7.5% from the stock solution)	7.712 ± 2.736
10× (10% from the stock solution)	2.875 ± 2.815
8× (12.5% from the stock solution)	0
6.7× (15% from the stock solution)	0

**Table 2:** Minimum Inhibitory Concentration (MIC) and Minimal Microbial Concentration (MMC) values of ozonized oil in liposomes (Ozodrop^®^) in relation to reference antibiotics.

Microorganisms	Ozodrop		Gentamicin	Tobramycin	Levofoxacin	Itraconazole
	MIC (μg/mL)	MMC (μg/mL)	MIC (μg/mL)			
*S. aureus* ATCC 29213	32	62.5	0.5	0.5	0.06	-
*S.aureus* ATCC 43300 (MRSA)	32	125	0.25	0.25	0.125	-
*S. epidermidis* ATCC 14990	62.5	125	0.03	0.06	0.015	-
*M. catarrhalis*	62.5	125	0.25	0.5	0.06	-
*P. aeruginosa* ATCC 27683	62.5	125	0.5	0.25	0.5	-
*E. coli* ATCC 25922	62.5	125	1	1	0.015	-
*C. albicans*	32	62.5	-	-	-	1

Abbreviations: MIC, Minimum Inhibitory Concentration; MMC, Minimal microbicidal concentration; *S. aureus, Staphylococcus aureus; S. epidermidis, Staphylococcus epidermidis; M. catarrhalis, Moraxella catarrhalis; P. aeruginosa, Pseudomonas aeruginosa; E. coli, Escherichia coli; C. albicans, Candida albicans*; and MRSA, Methicillin-Resistant *Staphylococcus aureus*. “-”—indicates no detectable activity.

## Data Availability

The data that support the findings of this study are available from the corresponding author, R.P., upon reasonable request.
